# A Case of Edwardsiella tarda Infection With Iliopsoas Abscess Following Acute Pyelonephritis

**DOI:** 10.7759/cureus.58868

**Published:** 2024-04-23

**Authors:** Hiroyuki Matsukawa, Daisuke Usuda, Hiroki Takami, Tomohisa Nomura, Manabu Sugita

**Affiliations:** 1 Department of Emergency and Critical Care Medicine, Juntendo University Nerima Hospital, Tokyo, JPN

**Keywords:** case report, treatment, bacteremia, pyelonephritis, iliopsoas abscess, edwardsiella tarda

## Abstract

*Edwardsiella tarda *(*E. tarda*) is a gram-negative bacillus commonly isolated from aquatic environments and various aquatic animals. It rarely causes infections in humans, but rare human infections occur primarily through ingestion of infected seafood or aquatic animals. Symptoms include fever, gastroenteritis, and diarrhea, but severe extraintestinal infections have also been reported. This report describes a 76-year-old female developing *E. tarda* infection with iliopsoas abscess following acute pyelonephritis. Her chief complaint was fatigue and difficulty moving. Blood tests showed an increased inflammatory response, but the cause could not be identified from the patient's medical history, physical findings, and imaging findings. We diagnosed it as a urinary tract infection from the results of gram staining and started treatment, but the fever persisted thereafter, and a contrast-enhanced CT scan performed for re-evaluation revealed an iliopsoas abscess. After CT-guided abscess drainage, the patient made good progress and was transferred to a rehabilitation hospital on day 48 of the presentation. To the best of our knowledge, this is the first report of a case of *E. tarda *infection with iliopsoas abscess following acute pyelonephritis. Iliopsoas abscess is often difficult to diagnose. In this case report, we also present how we diagnosed and treated iliopsoas abscesses.

## Introduction

*Edwardsiella tarda* (*E. tarda*) is an Enterobacteriaceae-family bacterium that was first reported in 1965, by Ewing [1]. The pathogen bacillus is motile, facultatively anaerobic, and gram-negative, and is oxidase-negative and catalase-positive [1-4]. It is commonly isolated from aquatic environments and various aquatic animals (such as reptiles, amphibians, and fish, including catfish and eels); it only rarely causes infections in humans [2,3,5-10]. These rare human infections occur primarily through oral ingestion of infected seafood or aquatic animals, and present with a fever, gastroenteritis, and diarrhea [2-6]. However, severe extraintestinal infections have also been reported, including bacteremia, wound infections, necrotizing fasciitis, hepatobiliary infections (liver abscess and cholecystitis), peritonitis, meningitis, osteomyelitis, salpingitis, endocarditis, urinary tract infection, tubo-ovarian abscess, psoas abscess, epidural abscess, brain abscess, and empyema [2-4,6-16]. The risk factors for extraintestinal infections are age >65 years, immunocompromised status, and subacute and chronic diseases, including hepatobiliary diseases, malignancy, and diabetes mellitus [2,4,5,8,10,13]. Other factors associated with increased *E. tarda* extraintestinal infection risk include ingestion of raw fish, exposure to aquatic environments, and iron overload states (sickle cell disease, leukemia, and neonatal state) [2,4,13,14].

As mentioned, the various extraintestinal infections above have been reported to date, but there have been no reports of *E. tarda* infection with iliopsoas abscess following acute pyelonephritis. Therefore, we report the first such case, together with a brief review of the literature.

## Case presentation

A 76-year-old Japanese woman presented to our hospital with a 15-day history of fatigue, which gradually worsened. Three days before the presentation, she had developed a fever of 38℃ and anorexia. She subsequently developed back pain and difficulty moving and was rushed to our hospital. She had not visited any other hospital for these symptoms before the presentation. She had a history of a right femoral neck fracture and cervical spondylosis, but no other medical history including diabetes mellitus, hypertension, and malignancy. She had undergone right bipolar hip arthroplasty for a right femoral neck five years earlier but had no other surgical history. She regularly took acetaminophen for cervical spondylosis for a year, but no other long-term medications. She also had not been prescribed antibiotics for at least six months. She reported no use of tobacco or illicit drugs, no habit of drinking alcohol, and no known allergies. Although she regularly ate raw fish, she had not consumed undercooked seafood or been exposed to aquatic environments in a week prior to becoming ill. The patient had no family history of hereditary diseases or malignant diseases. She was a homemaker and independent in her everyday life before the illness.

The patient was 143 cm tall and weighed 40.3 kg (body mass index: 19.7). On presentation, her temperature was 36.8℃ (she had taken acetaminophen two hours earlier); her blood pressure was 105/69 mm Hg, her heart rate was 86 beats per minute, her respiratory rate was 16 breaths per minute, her oxygen saturation was 92% while breathing ambient air, and her Glasgow Coma Scale score was 15 points (E4V5M6). There was tenderness in her left lower back, but the remainder of the physical examination showed normal results. She also had no history of dysuria, changes in urinary frequency, or gross hematuria that would suggest a urinary tract infection.

Her white cell count was 17,700/μL (reference range: 3,600-8,900/μL) and her C-reactive protein was 35.56 mg/dL (reference range: 0.00-0.29 mg/dL) (Table 1). A routine laboratory examination, taken upon arrival at the emergency department, also revealed increased values for total bilirubin, alkaline phosphatase, lactate dehydrogenase, γ-glutamyl transpeptidase, blood urea nitrogen, creatinine, and lactate (Table 1). On the other hand, her platelet count was decreased (Table 1). A urine qualitative test revealed positive results for white blood cells, occult blood, and protein.

**Table 1:** Laboratory findings and venous blood gas analysis on presentation

Parameter (unit)	Result	Reference range
White blood cell (/μL)	17,700	3,600-8,900
Stab cell (%)	7	0-18
Segmented cell (%)	89	22-72
Eosinophil (%)	0	1-9
Basophil (%)	0	0-2
Lymphocyte (%)	1	25-48
Mono (%)	3	2-12
Red blood cell (×10^4^/μL)	440	380-504
Hemoglobin (g/dL)	13.5	11.1-15.2
Hematocrit (%)	40.1	35.6-45.4
Blood platelet (×10^4^/μL)	3.1	15.3-34.6
Total bilirubin (mg/dL)	1.3	0.4-1.2
Aspartate aminotransferase (IU/L)	29	5-37
Alanine aminotransferase (IU/L)	31	6-43
Lactate dehydrogenase (U/L)	266	124-222
Alkaline phosphatase (U/L)	598	38-113
Gamma-glutamyl transpeptidase (IU/L)	708	0-75
Creatinine kinase (U/L)	141	47-200
Blood urea nitrogen (mg/dL)	52	9-21
Creatinine (mg/dL)	3.91	0.5-0.8
Na (mmol/L)	137	135-145
K (mmol/L)	4.3	3.5-5.0
Cl (mmol/L)	99	96-107
Blood gas analysis (ambient air)		
pH	7.419	7.35-7.45
Anion gap (mmol/L)	10.4	12-16
Lactate (mmol/L)	2.0	0.5-1.5

**Figure 1:**
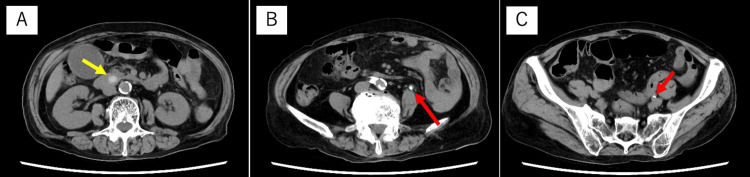
Thoracic and abdominal plane computed tomography scan on presentation A: A common bile duct stone 12 mm in size was confirmed (yellow arrow). There was no hydronephrosis; B: Multiple ureteral stones with a maximum diameter of 6 mm were found in the left ureter (red arrow); C: Multiple ureteral stones with a maximum diameter of 6 mm were found in the left ureter (red arrow).

**Figure 2:**
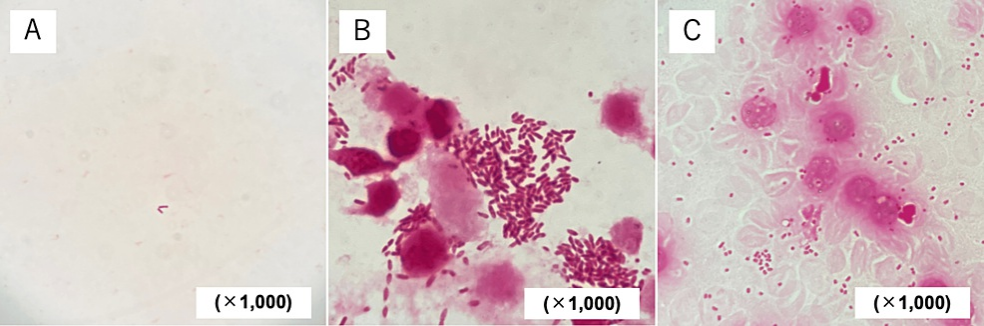
Microscopic findings on gram stains of bile, urine, and blood taken at the emergency department A: Gram stain of bile (×1,000). A few Gram-negative bacilli could be observed, but no white blood cells could be observed; B: Gram stain of urine (×1,000). Numerous Gram-negative bacilli and white blood cells were observed. A lot of intercellular bacilli were also seen within neutrophils, suggesting phagocytosis by white blood cells; C: Gram stain of blood (×1,000). Numerous Gram-negative bacilli and leukocytes were observed. Since this was a blood culture, many red blood cells were also observed. Some bacilli were seen within neutrophils, suggesting that they may be phagocytosed by leukocytes.

The cause of the high inflammation findings was still unclear from the medical history and physical findings, so we decided to perform a thoracic and abdominal computed tomography (CT) scan. Blood tests revealed severe renal dysfunction, so we decided not to use a contrast agent. A thoracic and abdominal plane CT scan showed a common bile duct calculus and multiple urinary calculi (Figure 1). At this point, we diagnosed acute cholangitis even though there was no right upper quadrant pain, because we found a common bile duct stone 12 mm in size, with the potential to be a cause of cholangitis, in addition to systemic inflammatory findings and elevated biliary enzymes. Because we observed renal dysfunction and thrombocytopenia, we considered severe acute cholangitis and performed an emergency endoscopic treatment after administering 0.5 g of meropenem (the dosage was adjusted to 0.5 g every 12 hours depending on the renal function). However, contrary to expectations, gram staining of bile revealed almost no bacteria and leukocytes (Figure 2a). On the other hand, numerous gram-negative bacilli and leukocytes were found in the patient’s urine (Figure 2b), leading to a diagnosis of left obstructive pyelonephritis. Results of blood cultures taken from bilateral median cubital veins were obtained after admission, and gram-negative bacilli were detected from both sets of blood cultures, indicating that pyelonephritis led to bacteremia. The causative bacterium was later identified as *E. tarda*, based on blood (using BD BACTEC^TM^ FX system, Becton, Dickinson and Company, United States) and urine cultures (using Phoenix^TM^ M50, Becton, Dickinson and Company, United States) (Figure 2c). Antimicrobial susceptibility testing of the pathogen is shown in Table 2.

**Table 2:** Result of antimicrobial susceptibility testing of blood culture Antimicrobial susceptibility was determined by the broth microdilution method. Breakpoints comply with the Clinical and Laboratory Standard Institute (CLSI) guidelines.

Bacteria: *Edwardsiella tarda*		
Antibiotics	Interpretation	Minimum inhibitory concentration (μg/mL)
Ampicillin	Susceptible	<8
Ampicillin/sulbactam	Susceptible	<8
Piperacillin	Susceptible	<8
Piperacillin/tazobactam	Susceptible	<16
Cefazolin	Susceptible	<4
Cefaclor	Susceptible	<8
Cefotiam	Susceptible	<8
Cefmetazole	Susceptible	<8
Flomoxef	Susceptible	<8
Cefoperazone/sulbactam	Susceptible	<16
Cefotaxime	Susceptible	<1
Ceftazidime	Susceptible	<4
Ceftriaxone	Susceptible	<1
Cefepime	Susceptible	<2
Cefcapene pivoxil	Susceptible	<0.25
Imipenem/cilastatin	Susceptible	<1
Meropenem	Susceptible	<1
Aztreonam	Susceptible	<4
Gentamicin	Susceptible	<2
Amikacin	Susceptible	<4
Minocycline	Susceptible	<2
Levofloxacin	Susceptible	<0.5
Fosfomycin	Susceptible	<4
Sulfamethoxazole-trimethoprim	Susceptible	<2

**Figure 3:**
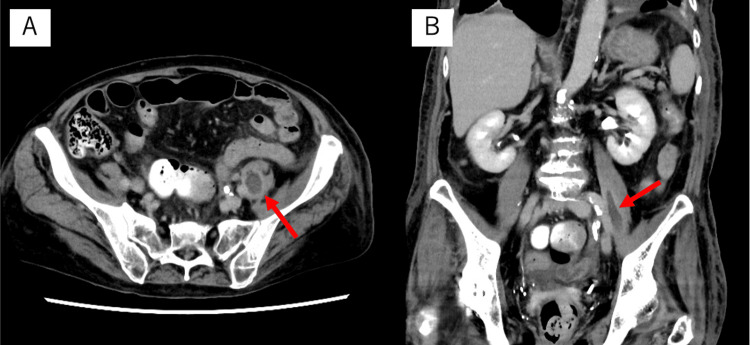
Abdominal contrast-enhanced computed tomography scan taken on day six of presentation A: Axial view. A low-density area with marginal contrast enhancement was observed inside the left iliopsoas muscle, and abscess formation was suspected (red arrow); B: Coronal view. A low-density area with marginal contrast enhancement was observed inside the left iliopsoas muscle, and abscess formation was suspected (red arrow).

**Figure 4:**
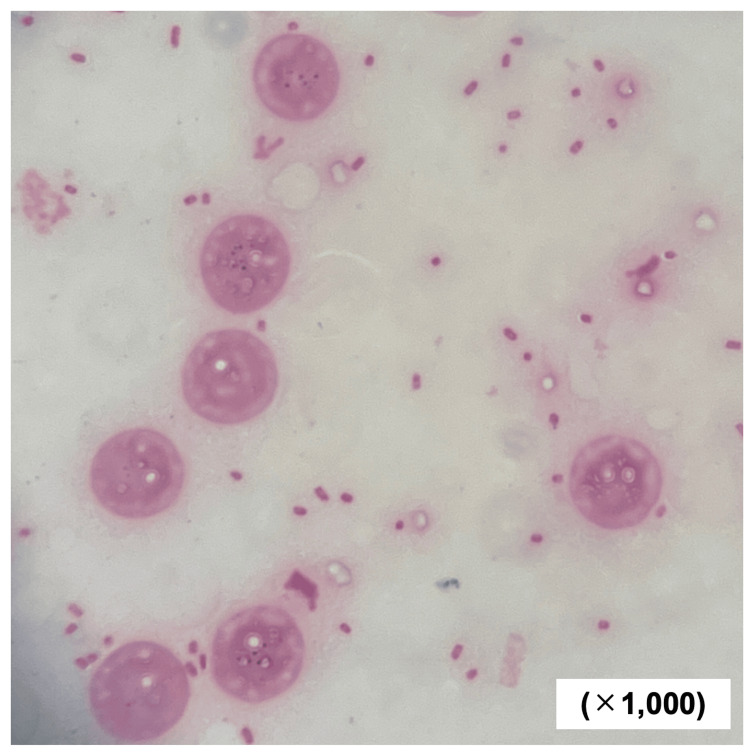
Microscopic findings of gram staining of iliopsoas abscess aspirate performed on day seven of presentation Numerous Gram-negative bacilli and leukocytes were observed in the gram stain of iliopsoas abscess aspirate. A lot of intercellular bacilli were also seen within neutrophils, suggesting phagocytosis by white blood cells.

All of the patient’s multiple ureteral stones were less than 6 mm in size and were expected to be excreted naturally [17], so we initially began treatment without placing a ureteral stent. However, the fever persisted even after the antibiotic was started, and 750 mg of acetaminophen was administered intravenously every 8 hours to 12 hours. There were no improvements in clinical findings, so a left ureteral stent was placed on day five of the presentation. Nevertheless, the fever continued, and a contrast-enhanced CT scan was performed on day six of the presentation. An abdominal contrast-enhanced CT scan disclosed a low-density area inside the left iliopsoas muscle, whose margin was contrast-enhanced (Figure 3). We diagnosed a left iliopsoas abscess, and CT-guided abscess drainage was performed on day seven of the presentation. Gram staining of the abscess aspirate revealed gram-negative bacilli, which was later identified as *E. tarda* (Figure 4).

The fever gradually began to subside around the day after abscess drainage. A culture test of the abscess aspirate fluid confirmed that the causative organism was *E. tarda* alone, so we changed the antibiotic to ampicillin (2 g every six hours) on day 10 of the presentation. A contrast-enhanced CT scan on day 18 of presentation to check the patient was being treated appropriately even after the de-escalation of antibiotics indicated that the abscess had shrunk, so on day 19 of presentation, the drain was removed, and the antibiotic was changed to amoxicillin (500 mg every eight hours) so that it could be managed without peripheral infusions. The patient continued to make good progress thereafter, but her muscle strength had weakened during hospitalization, so she was transferred to a rehabilitation hospital on day 48 of presentation after social adjustment. The clinical course of the patient is shown in Figure ​5. A contrast-enhanced CT scan on day 36 of the presentation was performed to confirm that the abscess continued to shrink even after antibiotics were changed from intravenous to oral administration. Antibiotic treatment was to be continued until the abscess disappeared or was immobilized based on imaging.

**Figure 5:**
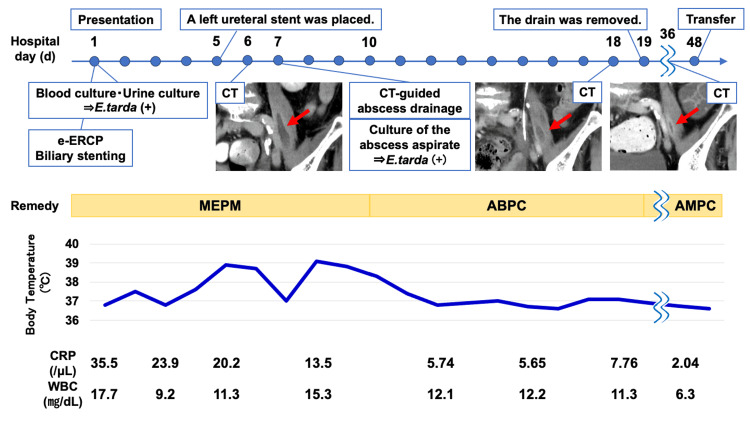
Clinical course of the patient Computed tomography performed on days 6, 18, and 36 were all contrast-enhanced. *E. tarda*: *Edwardsiella tarda*; e-ERCP: Emergency-endoscopic retrograde cholangiopancreatography; ABPC: Ampicillin; AMPC: Amoxicillin; MEPM: Meropenem; CRP: C-reactive protein

## Discussion

We report the first case of *E. tarda* infection with iliopsoas abscess following acute pyelonephritis. *E. tarda* is a gram-negative bacillus that lives in aquatic environments and is thought to rarely infect humans [1-4]. The rare infections in humans occur primarily through oral ingestion of infected seafood or aquatic animals [2-6]. Extraintestinal infections such as cholecystitis, urinary tract infections, meningitis, and necrotizing fasciitis have also been reported [2,14,16], but this is the first report of iliopsoas abscess resulting from a urinary tract infection.

She had some of the reported risk factors for extraintestinal infection, including age, hepatobiliary disease, and a history of raw fish consumption [2,4,5,8,10,13]. However, we assume that a common bile duct stone was unlikely to have been an influence as a risk factor for extraintestinal infection because it is believed that the infection was not transmitted through the biliary tract in this case. 

Transmission of water-loving organisms such as *E. tarda* and *Pseudomonas oryzihabitans* may not always involve contact with water or one-to-one contact with aquatic organisms carrying these organisms. For example, a case of *P. oryzihabitans* has been reported after tooth extraction, although there was no previous contact [18]. However, she had not received any dental treatment such as tooth extraction in at least six months prior to her illness. She continued to visit the hospital for cervical spondylosis, but she also had not undergone any invasive tests or treatments other than blood tests in the year before the illness. Previous studies have reported that *E. tarda* is highly lethal in non-intestinal infections [9], particularly in hosts who are immunocompromised, such as patients who have malignancies or diabetes, or who are currently taking immunosuppressive drugs for autoimmune diseases [19], though these claims remain controversial. Several studies have indicated an *E. tarda* bacteremia mortality rate of anywhere from 22.7% to 44.6% [4,5,13], but a group at Kurashiki Central Hospital in Okayama, Japan reported an overall 30-day mortality for *E. tarda* bacteremia of 12% and an overall 90-day mortality of 27% [8]. Although *E. tarda* demonstrates β-lactamase production, it has rarely been seen to be resistant to β-lactam antibiotics [9] and is in fact susceptible to most antimicrobial drugs, including tetracyclines, aminoglycosides, quinolones, antifolates, chloramphenicol, nitrofurantoin, and fosfomycin [8]. In this case, in particular, the pathogen demonstrated good susceptibility to most antibiotics (Table 2).

In this case, it was difficult to differentiate between pyelonephritis and acute cholangitis, but as expected, gram staining at the initial stages of treatment in the emergency department proved extremely important in diagnosis and determining the subsequent treatment strategy. Gram staining of bile did reveal a small number of gram-negative bacilli, but no white blood cells. It was true that *E. tarda* was also identified from bile culture, but when compared with gram staining of urine, it was clear which one should be more suspected as the source of infection. Since there was a common bile duct stone, it is possible that the flow of bile was blocked and *E. tarda* in the intestinal tract retrogradely invaded the bile, but we assessed that cholangitis did not develop and the gateway of *E. tarda* was the urinary tract.

Iliopsoas abscess can be broadly classified into primary iliopsoas abscess and secondary iliopsoas abscess based on its mechanism of occurrence [20]. Primary iliopsoas abscess occurs as a result of hematogenous or lymphatic spread of bacteria from distant sites [20]. Secondary iliopsoas abscess occurs due to direct bacterial expansion of the iliopsoas muscle from a nearby site of infection [20]. This case is considered to be a secondary iliopsoas abscess caused by direct infiltration from a urinary tract infection. *Staphylococcus aureus* and *Escherichia coli* are the most common causative organisms of primary and secondary iliopsoas abscesses, respectively [20]. Treatment of iliopsoas abscess involves appropriate antibiotics along with abscess drainage [20]. In this case, the patient developed an iliopsoas abscess, even though she had no underlying disease. This may have been due to her delay in coming to the hospital, as the acetaminophen she regularly took for her cervical spondylosis masked her symptoms. Patients like this one, who seemingly do not have any medical conditions that put them at risk of becoming serious cases, still run a risk of the course of the disease becoming unpredictably worse due to the masking of their clinical symptoms [18].

We were unable to diagnose the iliopsoas abscess from the beginning in this case. Broadly speaking, iliopsoas abscess frequently sees delayed diagnosis and delays in effective management, due to its tendency to present with varied, often non-specific symptomatology [20]. The so-called “classic triad” of iliopsoas abscess is back pain, limp, and fever [20]. We observed fever and lower back pain in this case, but we could not check whether she had a limp or not, because she had difficulty moving. Although we observed fever and back pain, these symptoms may also be observed in acute pyelonephritis. Therefore, it was unclear whether these symptoms suggested the presence of an iliopsoas abscess in this case.

Another limitation in diagnosis was the difficulty of performing a contrast-enhanced CT examination, due to the severe renal dysfunction observed at the initial blood test. Even when viewed retrospectively, a simple CT scan at the emergency department did not reveal any obvious lesions, so it is unclear whether an iliopsoas abscess existed from the start; even if it did exist, it would not have been possible to diagnose [21]. In this case, we were able to diagnose iliopsoas abscess by performing imaging tests, suspecting the presence of a mechanism for obstruction or abscess formation, because there were no improvements in the clinical findings even with appropriate antibiotic treatment. The diagnosis was also aided by the fact that her renal function improved markedly after admission without using hemodialysis, making it possible to perform a contrast-enhanced CT scan. Although urinary tract infections can cause secondary iliopsoas abscess [20], the clinical symptoms of acute pyelonephritis and those of typical iliopsoas abscess are similar, as demonstrated in this case. In order to diagnose complications of iliopsoas abscess, it is important to perform imaging tests at an appropriate time, based on clinical symptoms and inflammatory findings that fail to improve despite appropriate treatment.

## Conclusions

*E. tarda* infections in humans are considered to be relatively rare, and most of them are intestinal infections, but various extraintestinal infections, including this case, have also been reported and need attention. Iliopsoas abscess is a complication of urinary tract infections, but its diagnosis is often delayed. If there is insufficient improvement in clinical symptoms or laboratory findings despite appropriate treatment, it is important to consider the possibility of complications such as iliopsoas abscess and re-perform imaging tests.
